# miR-378a-3p regulates glioma cell chemosensitivity to cisplatin through IGF1R

**DOI:** 10.1515/biol-2021-0117

**Published:** 2021-11-02

**Authors:** Yunjiang Wang, Jia Du

**Affiliations:** Department of Neurosurgery, Yancheng Third People’s Hospital, Yancheng City, Jiangsu Province, 224001, China; Cancer Center, Daping Hospital, Army Medical University, No. 10 Changjiang Zhilu, Daping Yuzhong District, Chongqing, 400042, China

**Keywords:** glioma, miR-378a-3p, insulin like growth factor 1 receptor, cisplatin, chemosensitivity

## Abstract

Glioma is a type of common intracranial tumor. In this study, we investigated the molecular mechanism by which miR-378a-3p regulates cisplatin (CDDP) chemosensitivity in glioma cells via insulin-like growth factor 1 receptor (IGF1R). U251/CDDP cells were treated with CDDP and transfected with miR-378a-3p mimics, NC mimics, or pcDNA-IGF1R. qRT-PCR was used to measure the differential level of miR-378a-3p. CCK-8 assay was used to test cell proliferation, and flow cytometry was used to analyze apoptosis. The targeting relationship between miR-378a-3p and IGF1R was tested through a dual-luciferase reporter gene assay. In contrast to normal glial cells, the miR-378a-3p level decreased in human glioma U251 cells and had lower expression in U251/CDDP cells. Compared with the CDDP group, miR-378a-3p significantly caused the inhibition of U251/CDDP cell proliferation and enhanced apoptosis in the miR-378a-3p mimics + CDDP group. Another experiment confirmed that IGF1R was a target gene of miR-378a-3p, and overexpression of miR-378a-3p inhibited IGF1R expression. In addition, co-overexpression of miR-378a-3p and IGF1R induced the upregulation of the U251/CDDP cell proliferation and the inhibition of apoptosis in the miR-378a-3p mimics + pcDNA-IGF1R + CDDP group. This study confirmed that miR-378a-3p promoted the sensitivity of glioma cells to CDDP in glioma patients via targeting IGF1R to increase the therapeutic effect during chemotherapy.

## 1 Introduction

Glioma is an intracranial tumor, and patients experience increased intracranial pressure due to its mass effect in space, which can lead to vomiting, vision loss, psychiatric symptoms, or localized epilepsy [1]. Glioma can also result in limb pain and numbness, motor and sensory impairment, language expression, and understanding difficulties in patients due to the effect of glioma on the local brain tissue function [2]. Surgical resection is the main treatment for glioma, while chemotherapy and radiotherapy are important adjuvant therapies for glioma [2]. However, the therapeutic effect of chemotherapy is not ideal since cells from glioma patients are prone to develop drug resistance.

Cisplatin (CDDP) is an effective DNA alkylating agent and is the most common and effective chemotherapeutic drug in clinical use. It has a good therapeutic effect on many solid tumors [3]. However, prolonged use of CDDP can lead to drug resistance in humans. It has been reported that CDDP may cause nephrotoxicity or hepato-cardiotoxicity through oxidative stress, DNA damage, and inflammation [3,4]. Therefore, CDDP is of limited use due to its resistance and toxicity to nontargeted tissues. In order to solve the problem of drug resistance to CDDP in glioma patients, it is necessary to further explore the mechanisms underlying the drug resistance of CDDP in glioma and therefore strategically improve the chemosensitivity of glioma cells.

MicroRNAs (miRNAs) are small RNAs of highly conserved endogenous noncoding proteins that can bind to the 3′ untranslated regions (UTR) end of mRNAs for exerting their roles in negatively regulating gene expression [5]. miRNAs play an important regulatory role in drug resistance in various tumors, such as gastric cancer, cervical cancer, and particularly glioma [6,7]. Among them, miR-378a-3p is a suppressor of tumor cells such as colorectal cancer, lung cancer, and breast cancer [8,9,10]. However, the regulatory relationship between miR-378a-3p and the sensitivity of glioma cells to chemotherapy is unknown.

As a growth regulator, insulin-like growth factor 1 (IGF1) has a molecular structure similar to insulin and is an essential active substance for human growth [11]. IGF1 receptor (IGF1R) is able to mediate IGF-1 action, and the IGF1R signaling pathway is closely related to the occurrence, development, and metastasis of tumors, which is an important indicator for clinical cancer diagnosis and prognosis [12]. However, the specific effect of IGF1R on glioma is still unclear.

Therefore, the aim of the present study was to validate the involvement of IGF1R in the regulatory effects of miR-378a-3p on CDDP chemosensitivity in glioma cells.

## 2 Materials and methods

### 2.1 Culture of human astrocytes (HA) and human glioma cell U251

HA cells (Mingzhoubio, Ningbo, Zhejiang Province, China) and U251 cells (National Collection of Authenticated Cell Cultures, Shanghai, China) were cultured using Dulbecco’s modified Eagle’s medium (DMEM, Sigma, USA) containing 10% fetal bovine serum (FBS, Sigma, St Luis, MO, USA). The resistant cell line U251/CDDP was established by dose escalation screening of U251 cells in the logarithmic growth phase. For the next experiment, U251/CDDP cells were treated with 2 μg/mL CDDP (Med Chem Express, MCE, USA) and cultured for 24 h, and named as CDDP group.

### 2.2 Cell transfection

Cells at the logarithmic growth stage were taken, counted, and spread in 6-well plates. After cells were plastered, 4 μL (50 pmol/μL) of miR-378a-3p mimics was added to 100 μL of double-free medium (no serum, no antibiotics) and placed at room temperature for 5 min. The diluted miR-378a-3p mimics were mixed with 100 μL of Lipofectamine 2000 diluent and then added to 6-well plates and incubated at 37°C for 1 h. Next, the mixture was aspirated and then added to a complete medium (containing serum and antibiotics) and incubated for another 48 h.

### 2.3 Real-time fluorescence quantitative PCR assay (qRT-PCR)

RNA was extracted by using the Trizol method. cDNA was obtained according to the Reverse Transcription kit (Promega, Madison, WI, USA). The PCR system was prepared according to the instructions of the qRT-PCR kit (Promega). The reaction conditions were: 95°C for 30 s (pre-denaturation) and 40 cycles of amplification reaction (95°C for 5 s, 60°C for 20 s). The miR-378a-3p level was calculated by the 2^−∆∆Ct^ method and U6 was used as a reference. Primers for the reaction are listed in Table 1.

**Table 1:** Primers of qRT-PCR

Gene	Primers	Sequences (5′–3′)
miR-378a-3p	Forward	GCGCACTGGACTTGGAGTC
	Reverse	GCAGGGTCCGAGGTATTC
U6	Forward	CTCGCTTCGGCAGCACA
	Reverse	AACGCTTCACGAATTTGCGT

### 2.4 Cell Counting Kit-8 (CCK-8) assay for human glioma cell proliferation

U251 and U251/CDDP cells were treated with different concentrations of CDDP (0.1, 0.3, 0.9, 2.7, 8.1, 24.3, 72.9 and 218.7 μg/mL). Then, cells were added to 10% CCK-8 to the culture at 37°C for 1 h, and the absorbance values at a wavelength of 450 nm were measured. The formula for calculating the inhibitory rate of cell viability was: A value of control wells – A value of drug-administered wells/control wells × 100%, and the inhibitory concentration (IC_50_) of CDDP on cells was calculated from a linear regression of the logarithm of cell inhibition rate and drug concentration.

U251/CDDP cells were treated with CDDP for 24 or 48 h. Then, CCK-8 at a final concentration of 10% was added and incubated. The absorbance values at a wavelength of 450 nm were tested using a microplate reader, and the cells were analyzed to determine proliferative ability.

### 2.5 Detection of apoptosis in U251/CDDP cells by flow cytometry

U251/CDDP cells were treated with CDDP or transfection and then were digested and collected using 0.25% trypsin (Sigma) for 48 h. The digested cells were washed twice using 400 μL of pre-chilled 1× PBS, centrifuged, and the supernatant was discarded. The cells were resuspended with the binding buffer, added to 5 μL of Annexin V-FITC and 10 μL of propidium iodide (PI), and incubated for 10 min [13]. The apoptotic ratio of cells was surveyed by CyFlow^®^ Cube 8 flow cytometry (Sysmex-Partec, Germany).

### 2.6 Dual-luciferase reporter gene assay

Cells were co-transfected with IGF1R-3′ UTR wild-type (WT) or IGF1R-3′ UTR mut and miR-378a-3p mimics or NC mimics. Cells were digested with 0.25% trypsin, and the supernatant was then discarded after centrifugation. The luciferase activity was measured based on the Dual Luciferase Assay Kit (Zeye, Shanghai, China). The relative activity of luciferase was equal to the firefly luciferase activity value divided by the sea kidney luciferase activity value.

### 2.7 Western blotting

After CDDP treatment or transfection with miR-378a-3p mimics, NC mimics, pcDNA-IGF1R, and pcDNA, cells were digested and collected using 0.25% trypsin, washed twice using pre-chilled 1× PBS ( phosphate buffer saline), centrifuged, and supernatants were discarded. The total cellular protein was extracted and then separated by SDS-PAGE electrophoresis and transferred to the PVDF membrane. The cells were closed with 4% skim milk for 1 h, incubated overnight at 4°C with primary antibody dilution of IGF1R (ab182408, 1:1,500, Abcam, Cambridge, MA, USA), washed 3 times with 1× phosphate buffered saline Twen-20, and then incubated for 1 h using secondary antibody dilution (ab7090, 1:2,000, Abcam), and developed by adding ECL developer [14].

### 2.8 Statistical processing

The data were analyzed using Prism 8 software (GraphPad Software, Inc., San Diego, CA, USA), and the experimental results were expressed as mean ± standard deviation. The one-way analysis of variance (ANOVA) was used for data analysis among three or more groups, and the least significant difference *t*-test was performed when significant differences were determined. Differences were considered statistically significant at *p* < 0.05.

## 3 Results

### 3.1 miR-378a-3p was downregulated in drug-resistant U251/CDDP cells

**Figure 1:**
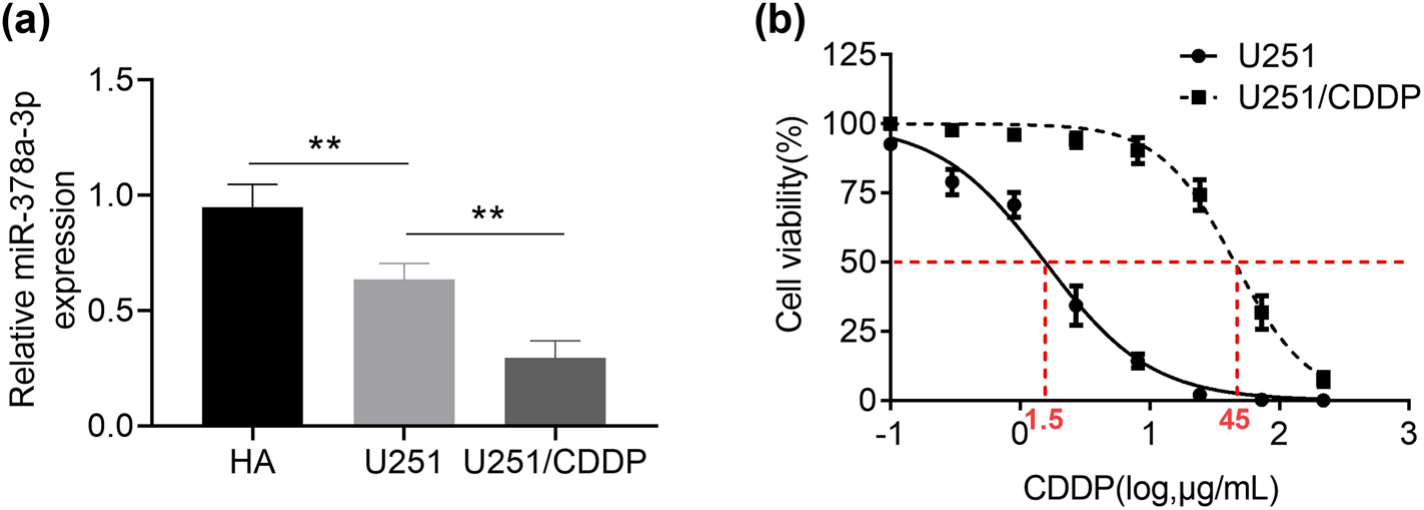
Expression of miR-378a-3p in drug-resistant U251/CDDP cells. (a) miR-378a-3p expression was detected by qRT-PCR. (b) Dose–response curve of CDDP administration on glioma cells was detected by the CCK-8 assay.^**^ *p* < 0.01.

The results in Figure 1a showed that miR-378a-3p expression was reduced in U251 cells (*p* < 0.01) compared with HA cells, while the lowest expression of miR-378a-3p was seen in U251/CDDP cells (*p* < 0.01). The resistance of U251 cells and U251/CDDP cells to CDDP was examined under different concentration gradient conditions. The results of the CCK-8 assay in Figure 1b showed that the IC_50_ for CDDP was 1.5 μg/mL in U251 glioma cells and 45 μg/mL in U251/CDDP cells, indicating the drug-resistant ability of U251/CDDP cells. Taken together, miR-378a-3p was downregulated in drug-resistant U251/CDDP cells, indicating its potential role in drug resistance.

### 3.2 Effects of miR-378a-3p on the chemosensitivity of U251/CDDP cells

**Figure 2:**
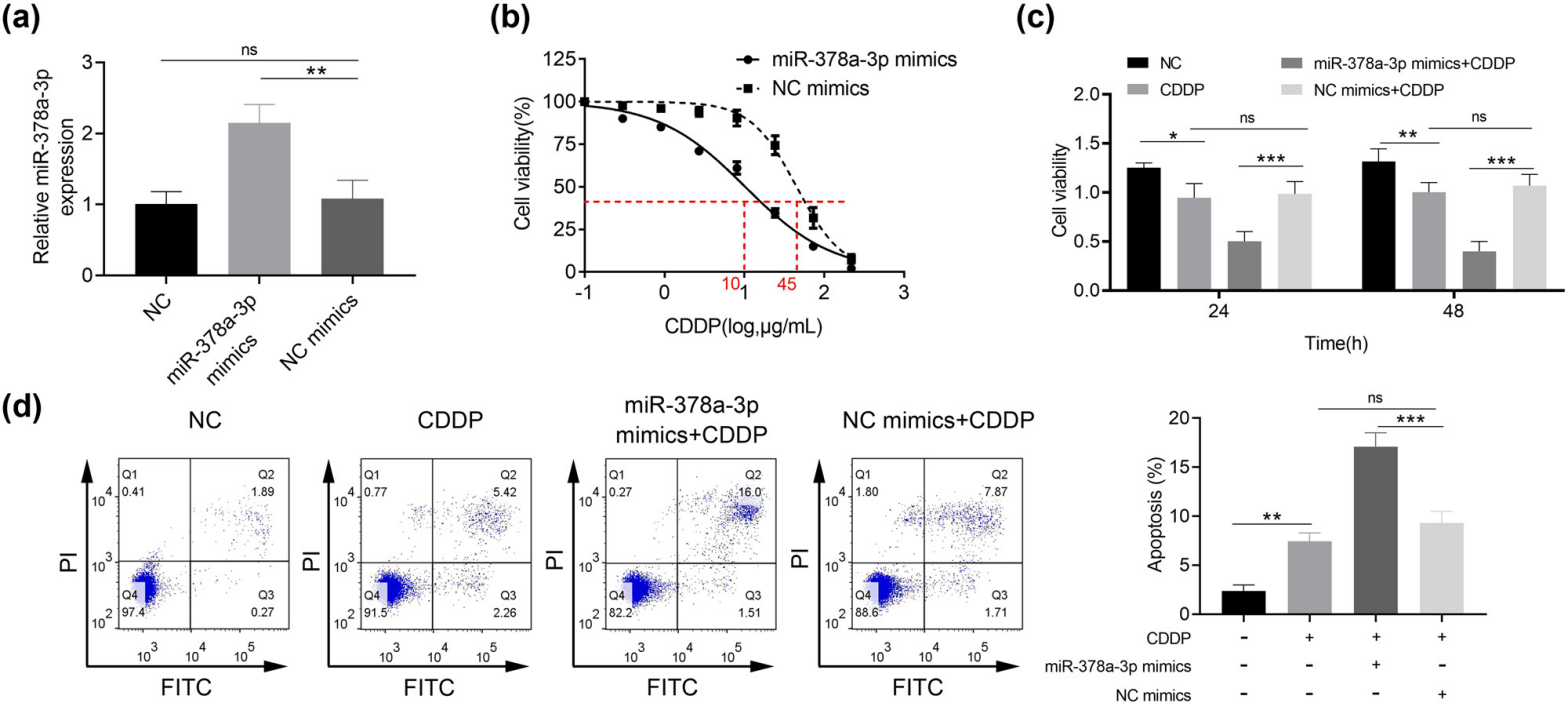
Effects of miR-378a-3p on the chemosensitivity of CDDP/U251 cells. (a) qRT-PCR assay was used to validate miR-378a-3p transfection efficiency. (b) Dose–effect curve of CDDP administration on glioma cells after transfection of miR-378a-3p/NC mimics in U251 cells. (c) The proliferation of U251/CDDP cells was detected by the CCK-8 assay. (d) The apoptosis of U251/CDDP cells was detected by flow cytometry. ^*^ *p* < 0.05, ^**^ *p* < 0.01, and ^***^ *p* < 0.001.

The qRT-PCR results in Figure 2a indicated that the level of miR-378a-3p was significantly increased in the miR-378a-3p mimics group as compared to NC mimics (*p* < 0.01), which suggested the successful transfection of miR-378a-3p mimics in U251/CDDP cells.

The degree of resistance to CDDP after transfection with miR-378a-3p/NC mimics in U251/CDDP cells was examined under different concentration gradient conditions. The results of the CCK-8 assay in Figure 2b showed that the IC_50_ for CDDP in the miR-378a-3p mimics group was 10 μg/mL, and the IC_50_ for CDDP in the NC mimics group was 45 μg/mL in U251/CDDP cells, indicating that miR-378a-3p mimics decreased the resistance of U251/CDDP cells to CDDP. In Figure 2c, the administration of CDDP suppressed the proliferation of U251/CDDP cells as compared to the control group ( *p* < 0.05 at 24 h, *p* < 0.01 at 48 h). Then, the proliferation of U251/CDDP cells was significantly inhibited in the miR-378a-3p mimics + CDDP group compared with the NC mimics + CDDP group ( *p* < 0.001). The results in Figure 2d revealed that CDDP administration induced U251/CDDP cell apoptosis ( *p* < 0.01). Then, the apoptosis rate of U251/CDDP cells was clearly increased in the miR-378a-3p mimics + CDDP group compared with the NC mimics + CDDP group ( *p* < 0.001). To sum up, miR-378a-3p could accelerate the inhibitory effects of CDDP on the proliferation and also aggravated the promotive effects of CDDP on the apoptosis of U251/CDDP cells.

### 3.3 miR-378a-3p targets and negatively regulates IGF1R

**Figure 3:**
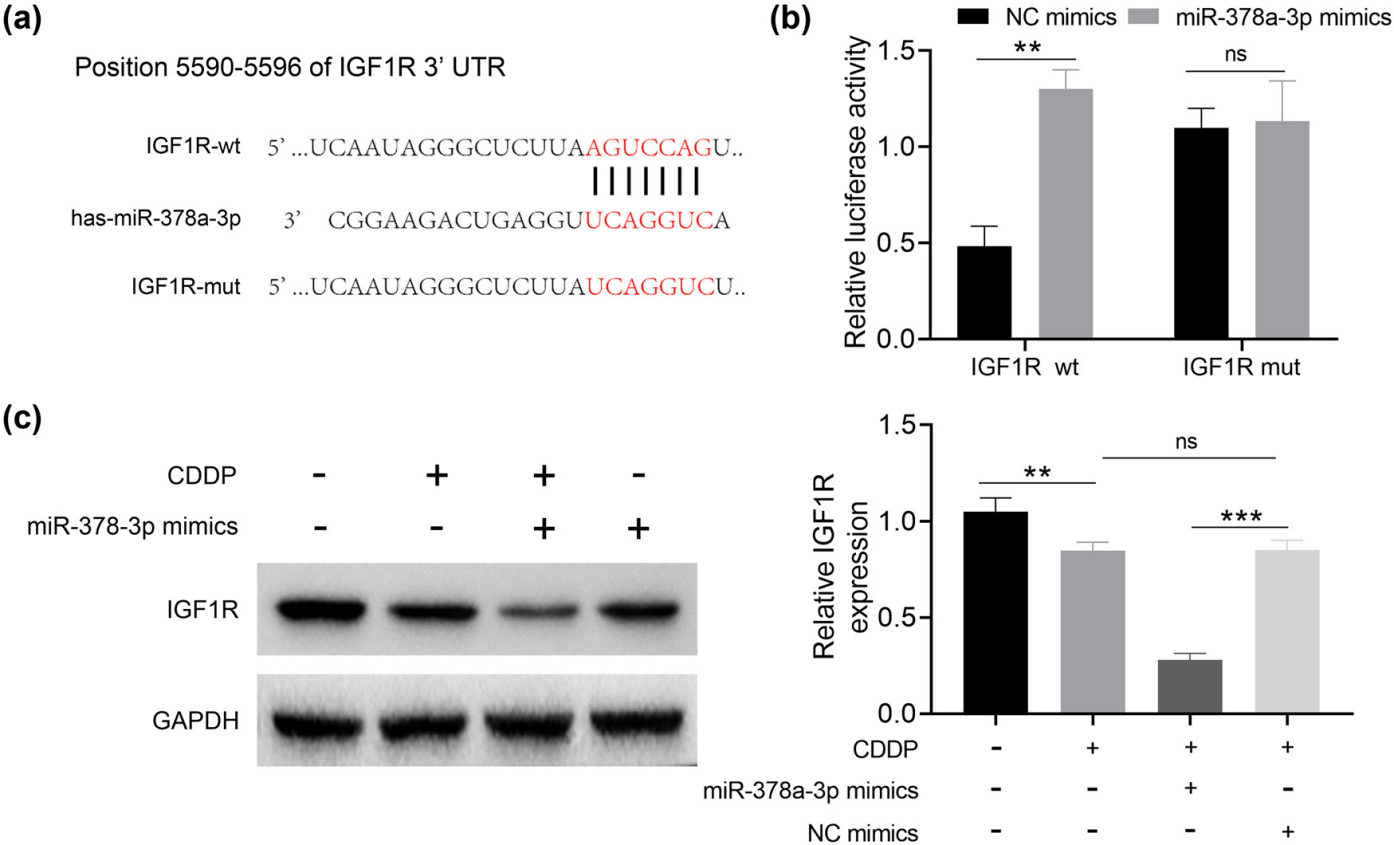
miR-378a-3p directly targets IGF1R. (a) Binding sequences between miR-378a-3p and IGF1R. (b) Dual luciferase reporter assay was used to detect the binding relationship between miR-378a-3p and IGF1R. (c) Western blotting was used to detect the protein expressions. ^**^ *p* < 0.01 and^***^ *p* < 0.001.

As shown in Figure 3a, the bioinformatics online analysis website miRanda and Targetscan predicted that IGF1R might be a target gene of miR-378a-3p. The results in Figure 3b showed that miR-378a-3p mimics remarkably inhibited the luciferase activity of IGF1R-wt (*p* < 0.01), while miR-378a-3p mimics had no inhibitory effect on the luciferase activity of IGF1R-mut, illustrating that miR-378a-3p could target IGF1R. Figure 3c displayed that IGF1R expression was reduced in the CDDP group compared to the control group (*p* < 0.01), while IGF1R expression was clearly decreased in the miR-378a-3p mimics + CDDP group as compared to that in the NC mimics + CDDP group (*p* < 0.001). Therefore, IGF1R was negatively regulated by miR-378a-3p.

### 3.4 miR-378a-3p promotes the chemosensitivity of CDDP/U251 cells by regulating IGF1R

**Figure 4:**
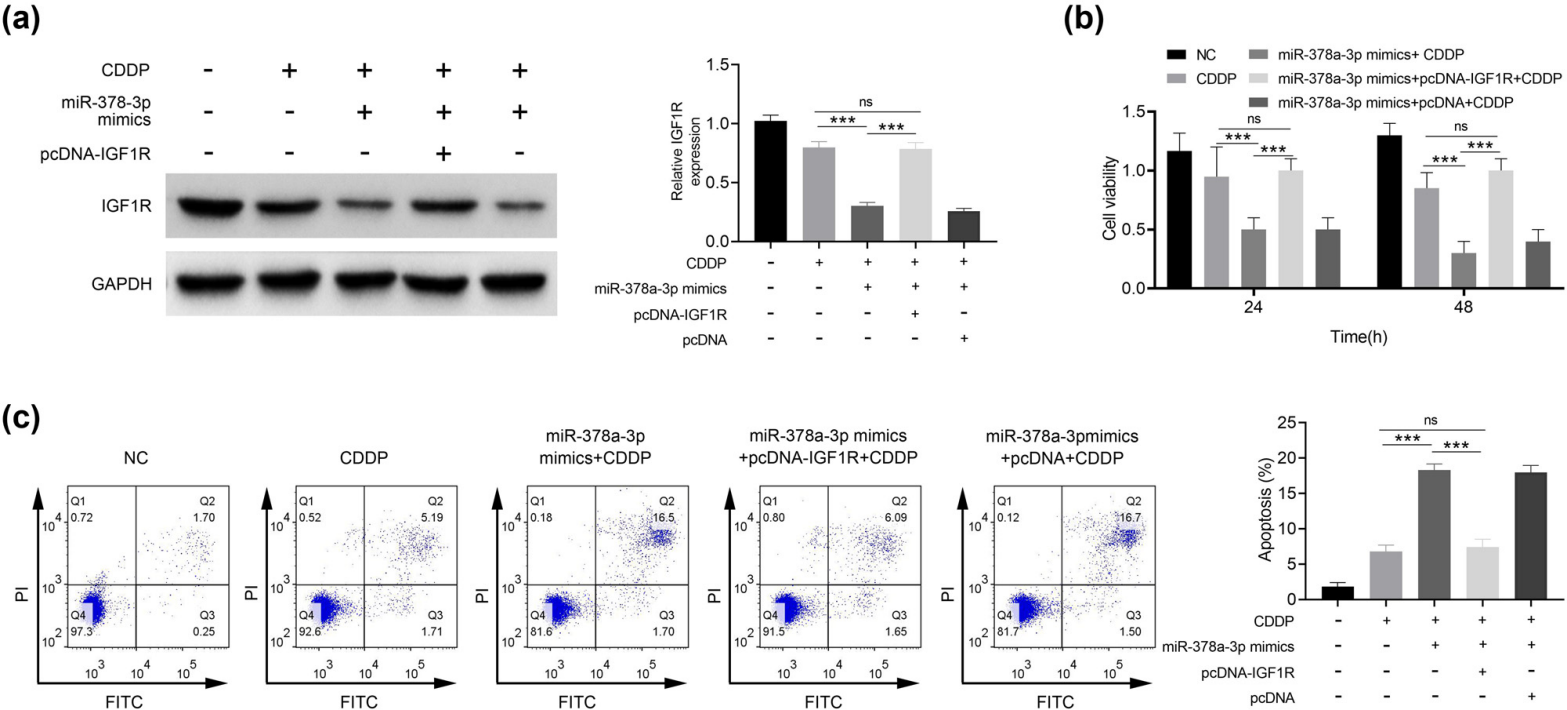
miR-378a-3p promotes the sensitivity of CDDP/U251 cells by regulating IGF1R. (a) IGF1R protein expression was detected by Western blotting. (b) Cell proliferation was detected by the CCK-8 assay. (c) Cell apoptosis was detected by Annexin V-FITC/PI and flow cytometry. ^***^ *p* < 0.001.

As shown in Figure 4a, IGF1R expression was lower in the miR-378a-3p mimics + CDDP group as compared to the control group (*p* < 0.001). IGF1R expression was increased in the miR-378a-3p mimics + pcDNA-IGF1R + CDDP group as compared to the miR-378a-3p mimics + pcDNA + CDDP group, indicating the successful transfection of IGF1R overexpression vector in U251/CDDP cells (*p* < 0.001). The results in Figure 4b and c showed that the proliferation was increased and the apoptosis was inhibited in U251/CDDP cells in the miR-378a-3p mimics + pcDNA-IGF1R + CDDP group as compared to the miR-378a-3p mimics + CDDP group (*p* < 0.001). Thus, the effects of miR-378a-3p on promoting apoptosis and inhibiting proliferation of U251/CDDP cells were reversed by IGF1R.

## 4 Discussion

Glioma accounts for about 80% of primary tumors in the human brain, which is highly malignant with a poor prognosis [15]. Currently, surgery is the mainstay of treatment supplemented by chemotherapy, radiotherapy, and immunotherapy to further improve the therapeutic effect [16].

CDDP is a widely used and effective broad-spectrum antitumor drug among platinum compounds and is used in the treatment of many tumors, such as glioma, cervical cancer, osteosarcoma, and human oral cancer [17,18]. However, drug resistance is also one of the main reasons for discontinuation during chemotherapy [19]. The reasons for CDDP resistance include immunity of the targeted cells to the drug component and expulsion of CDDP before it has a chance to damage the cancer cell DNA.

Hence, drug resistance limits the clinical application of CDDP and affects the treatment of glioma. Studies have reported that various compounds, such as ascorbic acid, lemon oil, and royal jelly [4,20,21], can improve the toxic effects on the kidneys of mice through antioxidant and oxidative effects. Therefore, the specific mechanism of drug resistance development in glioma needs to be further investigated to explore new therapeutic targets.

miRNAs are currently considered as most used regulators to investigate the mechanism of drug resistance. As an oncogenic factor, miR-378a-3p is able to participate in the development of various tumors. miR-378a-3p was lowly expressed in the development of breast cancer, lung cancer, and colorectal cancer [8,9,10]. Consistently, the present study illustrated that miR-378a-3p was lowly expressed in human glioma U251 cells and U251/CDDP cells. In addition, the degree of CDDP resistance in U251/CDDP cells was examined, and the results suggested that lower levels of miR-378a-3p in U251 cells may be associated with CDDP resistance. miR-378a-3p was found to be downregulated and inhibited the proliferation and cell cycle in colorectal cancer cells [22]. Besides, miR-378a-3p can downregulate MAPK1, thereby inhibiting CDDP sensitivity in ovarian cancer cells [23]. The present study revealed that the cell proliferative ability was inhibited, while the cell apoptosis was significantly increased in the miR-378a-3p mimics + CDDP group as compared to the CDPP group, demonstrating that miR-378a-3p could promote the sensitivity of U251/CDDP cells to chemotherapy.

The present study identified IGF1R as a target gene of miR-378a-3p. IGF1R belongs to the receptor-type tyrosine kinase family and is highly expressed in many tumor cells, such as glioma, breast cancer, gastric cancer, and lung cancer [12,24]. IGF1R has a potential mitogenic role in promoting cell proliferation, regulating malignant transformation of cells, protecting tumor cells from apoptosis and other biological functions [25]. Besides, miRNA-532 was shown to inhibit the development of colorectal cancer by directly targeting IGF1R to inhibit the PI3K/Akt pathway [26]. It was further confirmed that miR-378a-3p was able to target and downregulated IGF1R and thus aggravated the inhibitory effects of CDDP on the proliferative capacity of U251/CDDP cells.

In conclusion, miR-378a-3p can directly inhibit the growth of glioma cells and promote apoptosis by targeting IGF1R expression, thereby enhancing the sensitivity to CDDP. Therefore, miR-378a-3p can be used as a chemoresistant target and provide a new idea for the clinical treatment of glioma. However, we also found that miR-378a-3p could participate in the chemosensitivity of glioma cells to cisplatin by regulating multiple targets in previous studies. Therefore, in future studies, we will conduct more studies on other miR-378a-3p target genes to further understand the mechanism of miR-378a-3p in improving the sensitivity of glioma to CDDP.
